# Fabrication and Evaluation of Lambda-Cyhalothrin Nanosuspension by One-Step Melt Emulsification Technique

**DOI:** 10.3390/nano9020145

**Published:** 2019-01-23

**Authors:** Chunxin Wang, Bo Cui, Liang Guo, Anqi Wang, Xiang Zhao, Yan Wang, Changjiao Sun, Zhanghua Zeng, Heng Zhi, Hongyan Chen, Guoqiang Liu, Haixin Cui

**Affiliations:** Institute of Environment and Sustainable Development in Agriculture, Chinese Academy of Agricultural Sciences, Beijing 100081, China; wangchunxin0213@163.com (C.W.); cuibo@caas.cn (B.C.); guoliang01@caas.cn (L.G.); angelking521@163.com (A.W.); zhaoxiang@caas.cn (X.Z.); wangyan03@caas.cn (Y.W.); sunchangjiao@163.com (C.S.); zengzhanghua@caas.cn (Z.Z.); zhiheng6996@163.com (H.Z.); chenhongyan0103@126.com (H.C.); liuguoqiang@caas.cn (G.L.)

**Keywords:** lambda-cyhalothrin, nanoformulation, one-step emulsification, biological activity

## Abstract

Recent years have witnessed significant progress in nanotechnology and pesticide research in pest control and crop protection. There are more motivations to develop nanoformulations that are less harmful to environment than conventional formulations. The use of nanosuspension has been proposed as a novel formulation to process poorly soluble pesticides. In this study, the lambda-cyhalothrin nanosuspension (LCNS) was prepared in a melt emulsification method. The prepared nanosuspension had a mean particle size of 12.0 ± 0.1 nm and a polydispersity index of 0.279 ± 0.135. The smaller particle size and polydispersity confer better wettability, stability and bioavailability than conventional suspension concentrates. The excellent properties of the nanosuspension were attributed to the reduced particle size and the emulsification and dispersion of the surfactants. The LCNS eliminates the need for organic solvents and significantly reduces the amount of surfactant required. The simple production process of LCNS saves production and equipment costs. The results indicate that lambda-cyhalothrin nanosuspensions would have a broad application prospect in agricultural production systems.

## 1. Introduction

Pesticides as important agrochemicals have made tremendous contributions to agricultural development and food safety [1,2,3,4,5]. The production and use of pesticides must be considered from the aspects of pesticide efficacy, storage stability, safety and residues in the crops. Therefore, it is necessary to process new formulations to maximize the efficacy of pesticides.

Suspension concentrate (SC) is a new pesticide formulation used to disperse pesticides in aqueous media and can minimize both organic solvent use and dust pollution [6,7]. SC solves the problem of solubility and dispersion of water-insoluble pesticides. SCs belong to a thermodynamically unstable system, and the drug particles have spontaneous deposition. According to Stoke’s Equation (1):

(1) $$V\;=\;\frac{D^{2}(d_{1}\;-\;d_{2})g}{18\eta }$$

where, *V* is particle settling velocity (cm/s); *D* is the diameter of the disperse phase (cm); *d*_1_ is the density of the disperse phase (g/cm^3^); *d*_2_ is the density of the disperse medium (g/cm^3^); *η* is viscosity of disperse medium (g/cm·s). It can be seen from Stoke’s formula that the settling velocity of particles is directly proportional to the square of the diameter of particles. Therefore, the diameter of particles is the main factor, among many factors. Controlling the particle size of suspension is an important way to improve the stability of suspension dispersion system.

The use of nanosuspension has emerged in recent years as a new nanoparticulate drug delivery system [8]. Different saturation solubility of large and small particles in suspension will result in concentration gradient, which will further cause particle aggregation and precipitation. The particle size of nanosuspension is uniform, which can avoid the Ostwald ripening compared with microsuspension [9]. Therefore, nanosuspensions can not only increase the solubility and dissolution rate, but also improve the stability and enhance efficacy [10,11,12].

**Figure 1:**
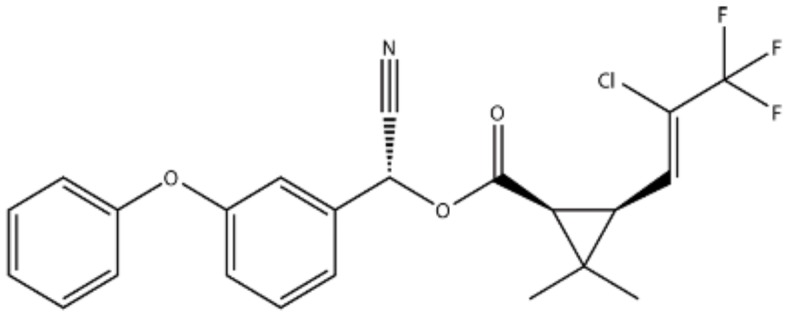
Chemical structure of lambda-cyhalothrin.

Most pesticide compounds have low solubility and poor dispersion in aqueous media [13]. Lambda-cyhalothrin (Figure 1) as a halogenated pyrethroid has a high and broad-spectrum, high efficiency and fast drug release. However, its water solubility is extremely low. According to the Ostwald-Freundlich equation, a smaller particle size would provide better dispersion and a larger specific surface area, which would further increase the dissolution rate and permeation [14,15,16]. Nanosuspensions are colloidal dispersions of nanoscale drug particles stabilized by surfactants and are typically prepared to increase the solubility of poorly water-soluble pesticides. There are many methods for preparing nanosuspensions such as wet milling, high-pressure homogenization, emulsion solvent evaporation, melt emulsification and supercritical fluid techniques [17,18,19].

In this study, a lambda-cyhalothrin nanosuspension (LCNS) was prepared using a one-step melt shearing emulsification method, which takes into account the low melting point of the pesticide. One-step shearing emulsification omits the complicated steps of high-pressure homogenization, which reduces the energy consumption and simplifies the preparation process. The hydrated mean particle size and polydispersity index (PDI) of the nanosuspension were measured by dynamic light scattering (DLS). The morphology of nanoparticles was observed by scanning electron microscopy (SEM) and transmission electron microscopy (TEM). The suspensibility, wettability and storage stability were characterized to demonstrate the particle size effect on the suspension properties. In addition, the insecticidal effect on aphids was evaluated to reveal the biological activity in this article.

## 2. Materials and Methods

### 2.1. Materials

Lambda-cyhalothrin (96%) was purchased from Changzhou Tianze Chemical Co., Ltd. (Changzhou, China). Alkylphenol formaldehyde resin polyoxyethylene ether (emulsifier 700), phenylethyl phenol polyoxyethylene polyoxypropylene ether (emulsifier 1601), styryl phenol polyoxyethylene ether (emulsifier 600), alkylphenol polyoxyethylene ether (OP-10) and nonylphenol polyoxy-ethylene ether (NP-7) were purchased from Cangzhou Hongyuan Agrochemical Co., Ltd. (Cangzhou, China). Maleic rosin polyoxypropylene polyoxyethylene ether sulfonate (MRES) was provided by Jiangsu Sinvochem S&D., Ltd. (Yangzhou, China). Sorbitan oleate (Span 80) (CAS number: 1338-43-8) and polyoxyethylene sorbitan fatty acid esters (Tween 80) (CAS number: 9005-65-6) were purchased from Shantou Xilong Chemical Co., Ltd. (Shantou, China). Lambda-cyhalothrin suspension concentrate (SC-A) was purchased from Sichuan Red Seed Agriculture Co., Ltd. (Chengdu, China). Lambda-cyhalothrin suspension concentrate (SC-B) was obtained from Guangdong Zhongxun Agricultural Science Co., Ltd. (Huizhou, China). HPLC-grade methanol was purchased from Fisher (Shanghai, China). Hard water (ρ (Ca^2+^ + Mg^2+^) = 342 mg/L) was obtained from China Agricultural University (Beijing, China) and Milli-Q water (18.2 MΩ. cm, total organic carbon ≤ 4 ppb) was used in all analytical experiments.

### 2.2. Methods

#### 2.2.1. Preparation Procedure of LCNS

LCNS was successfully prepared in a one-step emulsification method, which was adapted from the process described in the literature [20,21,22,23]. The emulsification process includes the following key stages: (1) 7.5 g lambda-cyhalothrin was dispersed in 80 °C deionized water. (2) 1.125 g emulsifier 700 was dispersed in deionized water, and added dropwise to lambda-cyhalothrin aqueous solution at 80 °C. (3) The mixture was emulsified in a high shearing machine (NANOJ H10, ATS, Shanghai, China) at 10,000 rpm for 5 min. During the emulsification process, the temperature was maintained at 80 °C to avoid premature solidification of the melt. The best mass ratio of pesticide/emulsifier 700 was typically 3:20. (4) Subsequently, the emulsion was cooled to ambient temperature with stirring at 600 rpm on a magnetic stirrer. Finally, a 5% lambda-cyhalothrin nanosuspension was obtained.

#### 2.2.2. Particle Size Distribution (PSD)

The samples were dispersed in deionized water for analysis. The mean particle size, 90% diameter percentile (D90) and polydispersity index (PDI) of the nanosuspension were determined by dynamic light scattering (DLS) (Zetasizer Nano ZS90, Malvern, UK) at 25 °C. PDI less than 0.3 meant a fairly narrow size distribution and good dispersion. All the data were measured in triplicate.

#### 2.2.3. Microscopic Morphology

Morphological evaluation of the nanosuspension was conducted by SEM (JSM-7401F, JEOL Ltd., Tokyo, Japan) with 3 kV voltage and 10 mA current. The SEM samples were prepared by diluting the suspensions to 0.025% (*w/w*) and subsequently spreading the dilution onto a cleaned silicon slice. The samples were dried at room temperature and sprayed with platinum for 40 s with a sputter coater (ETD-800; Beijing Elaborate Technology Development Ltd., Beijing, China). 

The morphology of the nanosuspension was characterized by TEM (HT7700, Hitachi Ltd., Tokyo, Japan) with 80 kV accelerating voltage. 2 μL diluted solution (25 μg/mL) were placed onto a carbon-coated copper grid and were dried at room temperature for TEM measurement.

#### 2.2.4. Suspensibility Measurement

Lambda-cyhalothrin nanosuspension (5.0 mL) was added into 250 mL standard hard water. Subsequently, the solution was reversed 30 times in 1 min, and then was placed in a constant temperature water bath (EMS-10, Changzhou Renhe Co., Ltd., Changzhou, China) at 30 ± 1 °C for 30 min. Finally, the pesticide content of 225 mL solution in upper layer and 25 mL solution in lower layer were measured. The initial content of the suspension was *m_0_* (g), and the content of 25 mL solution was measured as *m_1_* (g). The suspensibility (*W*) was calculated according to Equation (2):

(2) $$W(\%)\;=\;\frac{m_{0}\;{-\;m}_{1}}{m_{0}}\;\times \;\frac{10}{9}\;\times \;100$$

#### 2.2.5. Wettability Test

The contact angles on hydrophilic and hydrophobic leaf surfaces were determined with an OCA20 contact angle machine (JC2000D2M, POWEREACH, Shanghai, China). 5 µL diluted solution (0.1%, *w/w*) was dropped onto the leaves, and then the drop on the leaf was recorded after 5 s. The five-point fitting analysis method was used to measure the contact angle. All measurements were conducted in triplicate.

The retention was measured using an impregnation method [24,25,26]. The leaf was immerged fully into the aqueous solution (0.1%, *w/w*) for 15 s, and then was taken out of the solution. The weight of the leaf was measured before and after treatment. The area of the leaf was recorded by a portable leaf area meter (Yaxin-1241, Beijing Yaxinliyi Science and Technology Co., Ltd., Beijing, China). There were many replicates for each treatment and control. The retention was calculated according to Equation (3):

(3) $$R_{m}\;=\;\frac{M_{1}\;-\;M_{0}}{S}$$

where, *R*_m_ is the retention (mg/cm^2^), *M*_0_ is leaf mass before immersion (mg), *M*_1_ is the leaf mass after immersion (mg), and *S* is the leaf area (cm^2^).

#### 2.2.6. Storage Stability

The physical and chemical stability were evaluated after storage for 7 days at 0 °C and for 14 days at 54 °C, according to the product standard of pesticide suspension. The mean particle size and PDI of the aqueous solution were measured to assess the physical stability. The content of lambda-cyhalothrin was determined for chemical stability by high-performance liquid chromatography (HPLC, Agilent 1260, Agilent Ltd., Palo Alto, CA, USA) with a C18 column (5 µm, 4.6 × 150 mm) and ultraviolet detection (UV; 245 nm). The mobile phase was methanol/water (80:20, *v/v*) at 1.0 mL/min flow rate. The injection volume was 20 µL

#### 2.2.7. Bioassays

Lambda-cyhalothrin formulations were diluted into five different concentration gradients. Firstly, the leaves were immerged in the different concentration solutions for 10 s, and then leaves were placed in culture dishes and dried at room temperature. Secondly, the aphids were transferred to the treated leaves. Turnip aphids (*Mustard* *aphid*) were fed at 25 ± 2 °C and 75 ± 5% relative humidity. The hours of light and darkness were 16:8. Lastly, the mortality was counted after 48 h and the dates were analyzed using data processing system. Median lethal concentration (LC_50_), toxicity regression equation, correlation coefficient, 95% confidence limits were analyzed with variance and regression analyses.

#### 2.2.8. Statistical Analysis

The data obtained were analyzed using a one-factor analysis of variance (ANOVA) and Duncan’s multiple range tests via IBM SPSS Statistics 21 (SPSS 21.0., IBM Corp., Armonk, NY, USA). The statistical data was computed as mean ± standard deviation (SD). Least significant difference (LSD) was used to analyze data. A probability (*p*) of less than 0.05 means significant differences.

## 3. Results and Discussion

### 3.1. The Effect of Surfactants Types

The comparison of mean particle sizes and PDI values for eight samples prepared with different surfactants (seven nonionic surfactants and one anionic type surfactants) is shown in Table 1. In all these screening experiments, the mean particle sizes of nanosuspensions with Span 80, Tween 80, emulsifier 600 and emulsifier 1601 were more than 100 nm. In contrast, MRES, OP-10, NP-7 and emulsifier 700 reduced the mean particle size to less than 50 nm. However, the D90 of the nanosuspensions with MRES, OP-10 and NP-7 were near 200 nm. The results indicated that the particle size distribution was not uniform. PDI values less than 0.3 indicate a narrow size distribution [27]. The PDI of nanosuspensions with MRES, OP-10 and NP-7 was more than 0.4. These also suggested non-uniform particle size distributions. Emulsifier 700 can be adsorbed onto the hydrophobic pesticide nanoparticles surface, and polyoxyethylene chains form a hydrophilic adsorption layer on the surface of particles, which reduces the van Edward attraction between particles [28,29]. Emulsifier 700 can provide long hydrophobic chains to enhance spatial repulsive force to decrease the particle aggregation [30]. Above all, emulsifier 700 exhibited a more pronounced tendency to diffuse and stabilize emulsions against coalescence, which resulted in smaller final particle sizes. Therefore, emulsifier 700 was chosen for further research.

### 3.2. The Effect of Surfactants Dosages

Correct surfactant content plays an important role in stabilizing nanoparticles, especially in suspension systems [31,32,33,34]. Table 2 shows that the mean particle size and PDI decreased with increasing surfactant concentration (pesticide/surfactant from 20/1 to 20/3) until the surfactant content reached a certain level. At the pesticide/emulsifier ratio of 20/3 (*w/w*), the mean particle size was 12.0 nm and the particle size distribution was narrow (PDI = 0.279). It was assumed that the surfactant molecules achieved maximum surface coverage of the particles. In addition, the surfactant strengthened the steric resistance effect by forming an adsorption layer on the particle surface [35]. When the surfactant exceeded the optimum concentration, further addition of emulsifier probably entangled the pesticide in aqueous solution, which decreased the stability. In addition, most of the surfactants tended to induce bridging effects, which led to the aggregation and growth of the nanoparticles [36]. As a result, the content of emulsifier was lower than that used in conventional formulations, and a 20/3 ratio of pesticide/emulsifier was chosen for preparation of LCNS.

### 3.3. Microscopic Morphology Analysis

**Figure 2:**
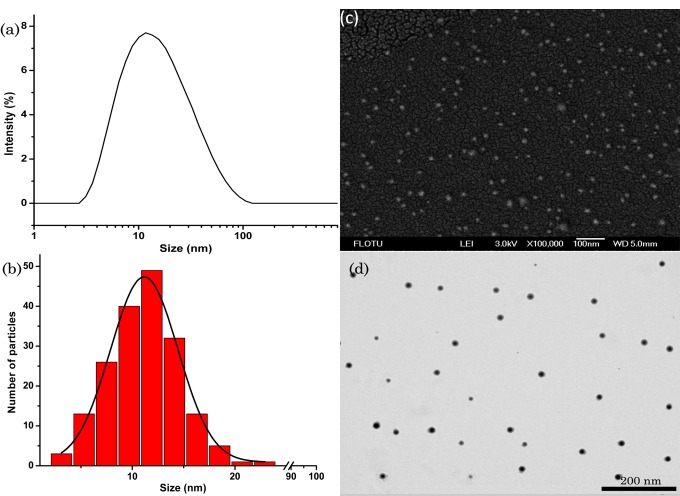
DLS and SEM characterization of the nanosuspension: (**a**) Particle size distribution based on DLS; (**b**) Particle size distribution based on SEM image; (**c**) SEM image of the lambda-cyhalothrin nanoparticles; (**d**) TEM image of the lambda-cyhalothrin nanoparticles.

SEM and TEM are useful tools to characterize particle morphology [37,38,39]. As shown in Figure 2a, the DLS image showed the size distribution based on the hydrodynamic diameter, and the mean particle size was 12.0 nm. The mean particle size based on the SEM image was 11.2 nm (Figure 2b), and the mean particle size based on the TEM image was 12.5 nm, which was consistent with the size measured by DLS. SEM image (Figure 2c) and TEM image (Figure 2d) showed that the nanoparticles were approximately spherical and uniform distribution.

### 3.4. Suspensibility

To investigate the kinetic stability of the suspensions, the suspensibility of the pesticide formulations was tested. The suspensibilities of SC-A and SC-B formulations were 84.9% and 87.8%, respectively. However, the suspensibility of the nanosuspension was 99.6%, which was higher than those of the commercial suspensions. This indicates that the kinetic stability of the nanosuspension was superior to the conventional formulations [40]. It was confirmed that suspensibility was inversely proportional to particle size, largely because brownian motion became acute with decreased particle size [41,42,43]. At the same time, surfactants also improved the pesticide dissolution performance. Above all, the excellent suspensibility of the nanosuspension was attributed to particle size reduction and the formulation’s composition.

### 3.5. Wettability and Retention

**Figure 3:**
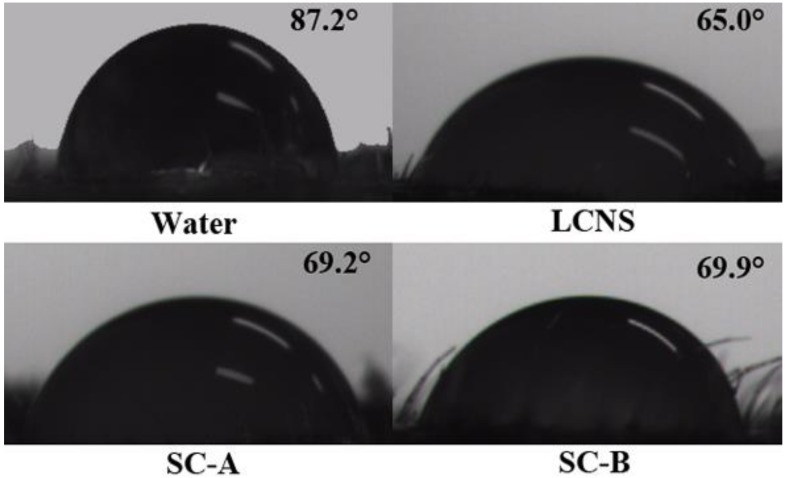
Contact angles image of different lambda-cyhalothrin formulations on cucumber leaves.

**Figure 4:**
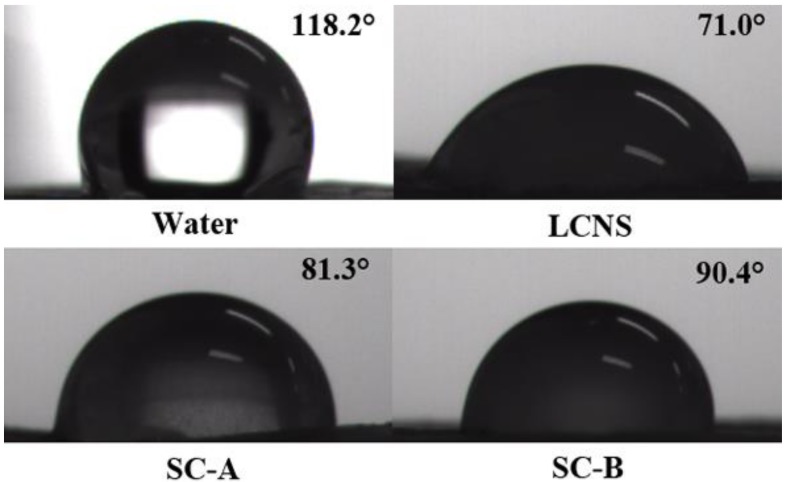
Contact angle image of different lambda-cyhalothrin formulations on brassica oleracea leaves.

**Figure 5:**
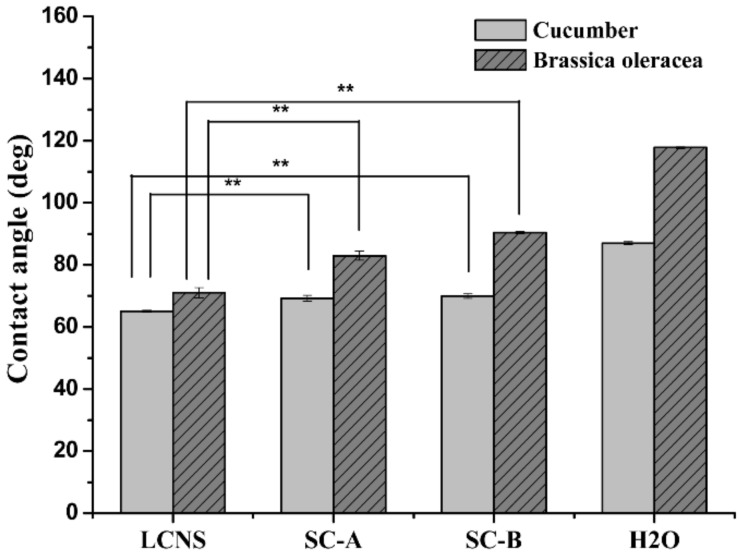
Contact angles of different lambda-cyhalothrin formulations on cucumber and brassica oleracea leaves (one-way ANOVA, followed by LSD test, ** *p* < 0.01).

The wettability is an important factor to assess the adsorption and adhesion capacity of pesticide on leaves. The contact angles of the formulation on cucumber (*Cucumis sativus* L.) and brassica oleracea (*Brassica oleracea* L.) leaves were measured and the results are presented in Figure 3 and Figure 4. As shown in Figure 5, the contact angles of the nanosuspension, SC-A and SC-B containing 0.1% (*w/w*) pesticide on cucumber were 65.0° ± 0.3°, 69.2° ± 0.9° and 69.9° ± 0.7°, respectively. Meanwhile, the contact angles of the nanosuspension, SC-A and SC-B containing 0.1% (*w/w*) pesticide on brassica oleracea leaves were 71.0° ± 1.6°, 81.3° ± 1.5° and 90.4° ± 0.3°, respectively. The result of a smaller contact angle indicated that the nanosuspension was easier to spread and wet on the leaf surface. As known to all, the surfactants can decrease surface tension, increase the diffusion of the solution, and further enhance the wettability on the leaves surface [44,45,46]. Besides, particle size reduction can increase the dissolution rate and supersaturation solubility [47,48].

**Figure 6:**
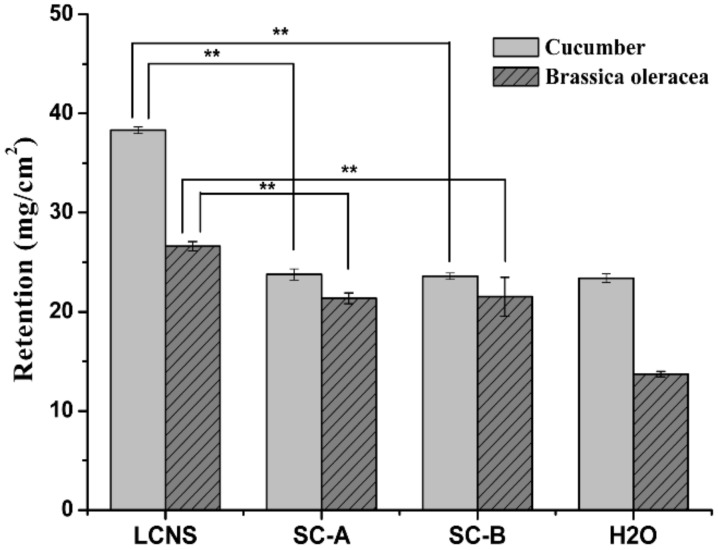
Retention of different lambda-cyhalothrin formulations on cucumber and brassica oleracea leaves (one-way ANOVA, followed by an LSD test, ** *p* < 0.01).

As shown in Figure 6, the retention of the SC-A and SC-B on cucumber leaves was 23.77 ± 0.56 and 23.59 ± 0.32 mg/cm^2^. The retention of the LCNS on cucumber leaves was 38.31 ± 0.32 mg/cm^2^, which was approximately 1.6 times that of the SC formulations. The retention of the SC-A and SC-B on brassica oleracea leaves was 21.37 ± 0.54 and 21.51 ± 1.96 mg/cm^2^. The retention of the LCNS on brassica oleracea leaves was 26.61 ± 0.46 mg/cm^2^, which was approximately 1.2 times that of the SC formulations. Indeed, the decrease of the particle size played a crucial role in expanding the particle surface area and contact area with the leaves [49]. Overall, these results reflect the excellent wettability of the LCNS.

### 3.6. The Stability of the LCNS

**Figure 7:**
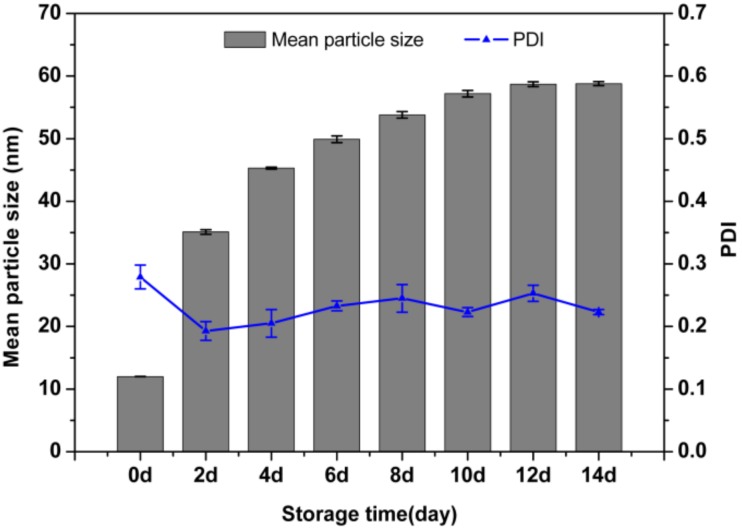
The mean particle size and PDI of the nanosuspension at 54 °C storage condition.

**Figure 8:**
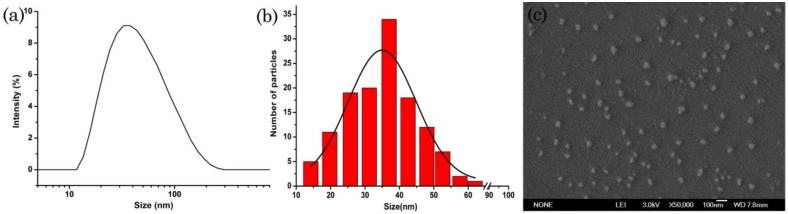
DLS and SEM characterization of the nanosuspension at 54 °C for 14 days: (**a**) Particle size distribution based on DLS; (**b**) Particle size distribution based on SEM image; (**c**) SEM image of the lambda-cyhalothrin nanoparticles.

In order to predict the performance change of product storage in the short term, mean particle size and PDI were measured to verify storage stability during storage at 0 °C for 7 days and 54 °C for 14 days. In Figure 7, the mean size increased to 58.5 ± 0.2 nm after storage at 54 °C for 14 days. The PDI was still below 0.3, which indicated a fairly narrow size distribution. As shown in Figure 8b, the mean particle size based on SEM image was 39.2 nm, which was smaller than the hydrodynamic size determined by DLS (Figure 8a). The result is consistent with literature reports that DLS gives a hydrodynamic size including the micelle core and the swollen corona, while SEM often gives the real particle size in a dried state [50,51]. Nanosuspensions are essentially thermodynamically unstable systems [52]. At a high storage temperature, the active drug particle may undergo Ostwald ripening, which caused the particles to adhere together and led to a comparative increase in particle size. It may be the main reason for aggregation and particle size increase [53,54]. A relatively slower rate of particle size increase was observed in this study with a proper concentration of stabilizers. By covering the surface of the nanoparticles, the surfactant molecules could shield the inner compound, decrease the free energy of the particles and reduce interfacial tension [55,56]. In addition, the polymeric structure of emulsifier 700 affords steric protection from agglomeration and prevents crystal growth [57,58,59]. As a result, particle size growth fluctuates little.

**Figure 9:**
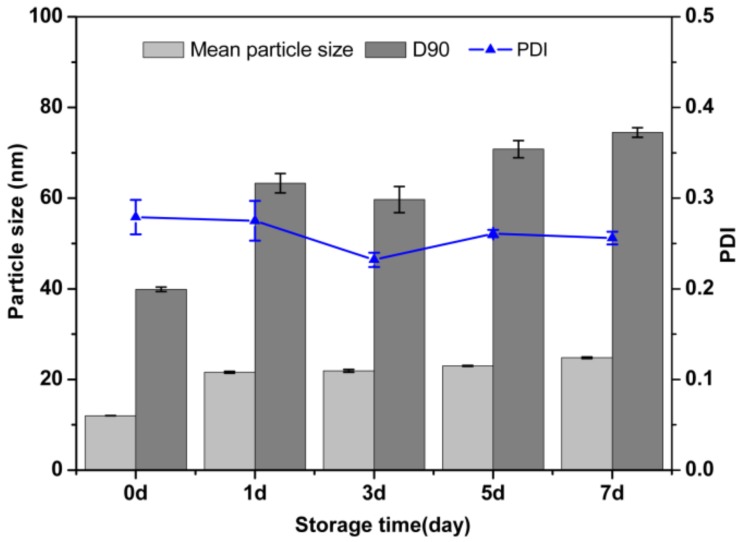
The mean particle size, D90 and PDI of the nanosuspension at 0 °C storage condition.

**Figure 10:**
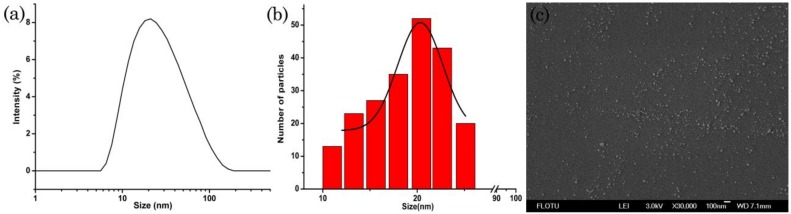
DLS and SEM characterization of the nanosuspension at 0 °C for 7 days: (**a**) Particle size distribution based on DLS; (**b**) Particle size distribution based on SEM image; (**c**) SEM image of the lambda-cyhalothrin nanoparticles.

In Figure 9, the mean particle size measured by DLS increased from 12.0 ± 0.1 nm to 24.8 ± 0.2 nm, and the PDI remained at 0.3 after storage at 0 °C for 7 days. In Figure 10b, the statistical mean particle size based on SEM image was 23.8 nm, which was consistent with the hydrodynamic size determined by DLS (Figure 10a). The Brownian motion of the particles weakened, and the aggregation degree of the particles decreased. Therefore, the change of particle size was relatively small. Overall, the nanosuspension showed good physical stability under different storage conditions.

The decomposition rate of lambda-cyhalothrin in nanosuspension was measured according to CIPAC MT 46 and GB/T 19136-2003. The decomposition of the lambda-cyhalothrin formulation was 0.83% after storage at room temperature, 2.25% after storage at 54 °C for 14 days and 2.99% after storage at 0 °C for 7 days. All of the decomposition rates met the standard of less than 5%, which indicates good chemical stability. Above all, it is apparent that the LCNS can still maintain excellent stability.

### 3.7. Biological Activity

In the study, the median lethal concentration (LC_50_) was used to evaluate insecticidal effects on *Mustard aphid*. Table 3 showed that LC_50_ of the LCNS was lower than that of the suspension concentrates [60,61]. The LC_50_ of the SC-B formulation was 1.7 times that of nanosuspension. Furthermore, the LC_50_ of the SC-A was much higher. These results suggest that LCNS had a better insecticidal effect than conventional suspension formulations. The decrease of the particle size increases the specific surface area of the particles, which makes the active components of pesticides easy to act on the biological target. In addition, nanoparticles have strong penetration and increase the absorption and accumulation of the drug particles by pests [62,63].

## 4. Conclusions

In this paper, we introduced a one-step emulsification method to prepare a lambda-cyhalothrin nanosuspension. The melt shearing emulsification method can solve the problem of poor solubility of insoluble pesticides, reduce the preparation cost and simplify the preparation process. The slow growth kinetics of the nanoparticles can be mainly attributed to the effect of emulsifier 700, which enhanced the stability of LCNS. Nanosuspension with uniform particle size below 100 nm has the characteristics of large specific surface area, fast dissolution rate and strong penetrability, and its effects are mainly manifested in improvement of solubility, wettability and insecticidal activity. Furthermore, the nanosuspension eliminated the need for organic solvents and reduced the required surfactant content. Therefore, lambda-cyhalothrin nanosuspensions could be expected to be a promising pesticide formulation in plant protection and ecological safety.

## Figures and Tables

**Table 1:** The effect of single surfactant on particle size and PDI of LCNS.

Surfactant	Mean Size (nm)	D90 (nm)	PDI
MRES	46.1 ± 0.5 de	209.3 ± 8.1 b	0.421 ± 0.003 b
OP-10	42.2 ± 0.4 e	223.3 ± 74.8 b	0.491 ± 0.037 b
NP-7	37.1 ± 0.2 e	188.0 ± 38.0 b	0.418 ± 0.029 b
Emulsifier 700	12.0 ± 0.1 e	39.9 ± 0.5 c	0.279 ± 0.135 b
Span 80	105.1 ± 4.1 cd	323.0 ± 154.8 b	0.332 ± 0.223 b
Emulsifier 600	140.4 ± 9.8 c	187.0 ± 41.5 b	0.441 ± 0.329 b
Emulsifier 1601	220.6 ± 13.8 b	247.3 ± 30.5 b	0.913 ± 0.150 a
Tween 80	565.5 ± 95.7 a	496.0 ± 119.4 a	1.000 ± 0.000 a

Different letters (a, b, c, d, e) at each data value indicate significant differences according to Duncan’s multiple range test at *p* < 0.05.

**Table 2:** The effect of surfactant concentration on the particle size and PDI of LCNS.

Ratio of Pesticide to Emulsifier 700	Mean Size (nm)	D90 (nm)	PDI
20/1	48.6 ± 0.8 a	207.6 ± 13.7 a	0.412 ± 0.016 a
20/2	22.2 ± 0.3 b	84.8 ± 3.4 b	0.357 ± 0.003 b
20/3	12.0 ± 0.1 e	39.9 ± 0.5 d	0.279 ± 0.019 d
20/4	16.8 ± 0.1 c	59.6 ± 5.4 c	0.313 ± 0.007 c
20/5	15.2 ± 0.1 d	50.8 ± 2.4 cd	0.279 ± 0.016 d

Different letters (a, b, c, d, e) at each data value indicate significant differences according to Duncan’s multiple range test at *p* < 0.05.

**Table 3:** Bioassay of three lambda-cyhalothrin formulations.

Formulation	Toxicity Regression Equation	Correlation Coefficient	LC_50_ (mg/mL)	95% Confidence Limit
LCNS	y = 0.8981x + 5.7229	0.9235	0.1566	0.0657–0.2697
SC-A	y = 1.1891x + 5.6716	0.9429	0.4177	0.3007–0.6487
SC-B	y = 1.0222x + 5.5773	0.9598	0.2724	0.1457–0.4145
